# Histological and biochemical evaluation of skeletal muscle in the two salmonid species *Coregonus maraena* and *Oncorhynchus mykiss*

**DOI:** 10.1371/journal.pone.0255062

**Published:** 2021-08-12

**Authors:** Bianka Grunow, Katja Stange, Ralf Bochert, Katrin Tönißen

**Affiliations:** 1 Institute of Muscle Biology and Growth, Leibniz Institute for Farm Animal Biology (FBN), Dummerstorf, Germany; 2 Mecklenburg-Vorpommern Research Centre for Agriculture and Fisheries (LFA MV) Institute of Fisheries; Research Station Aquaculture, Born, Germany; Universita degli Studi di Bari Aldo Moro, ITALY

## Abstract

The growth of fishes and their metabolism is highly variable in fish species and is an indicator for fish fitness. Therefore, somatic growth, as a main biological process, is ecologically and economically significant. The growth differences of two closely related salmonids, rainbow trout (*Oncorhynchus mykiss*) and maraena whitefsh (*Coregonus maraena*), have not been adequately studied as a comparative study and are therefore insufficiently understood. For this reason, our aim was to examine muscle growth in more detail and provide a first complex insight into the growth and muscle metabolism of these two fish species at slaughter size. In addition to skeletal muscle composition (including nuclear counting and staining of stem and progenitor cells), biochemical characteristics, and enzyme activity (creatine kinase, lactate dehydrogenase, isocitrate dehydrogenase) of rainbow trout and maraena whitefish were determined. Our results indicate that red muscle contains cells with a smaller diameter compared to white muscle and those fibres had more stem and progenitor cells as a proportion of total nuclei. Interestingly, numerous interspecies differences were identified; in rainbow trout muscle RNA content, intermediate fibres and fibre diameter and in whitefish red muscle cross-sectional area, creatine kinase activity were higher compared to the other species at slaughter weight. The proportional reduction in red muscle area, accompanied by an increase in DNA content and a lower activity of creatine kinase, exhibited a higher degree of hypertrophic growth in rainbow trout compared to maraena whitefish, which makes this species particularly successful as an aquaculture species.

## 1 Introduction

Maraena whitefish, (*Coregonus maraena*, Bloch 1779) and rainbow trout (*Oncorhynchus mykiss*, Walbaum, 1792) belong to the family of Salmonidae and are ecologically important for the Baltic Sea region and economically important for aquaculture production. At the beginning of the 20^th^ century, maraena whitefish was one of the most important food fish in the Baltic Sea region. A combination of extensive fishing, eutrophication of its estuary spawning areas, and habitat fragmentation through anthropic obstruction has brought the population of *C*. *maraena* to the edge of extinction. Intensive restocking operations started in the 1990s in Finland and later other Baltic Sea countries joined [1–3]. Through all these efforts the population of *C*. *maraena* could be stabilized, but is still on the red list as a vulnerable species [4]. Maraena whitefish is a species-rich genus of mostly cold-water benthivores that grow optimally at water temperatures between 13 and 18°C like it is found in the Baltic Sea [5]. Today this fish is also kept in aquaculture [6, 7]. The aim on the one side is to replenish the natural stocks of the Baltic Sea. On the other hand, these fishes will be marketed directly to the consumer. Because of this direct sale of *C*. *maraena*, our objective was to examine the skeletal muscles of this fish species in more detail at slaughter size under aquaculture conditions. The slaughter size and with it the carcass weight is fixed by the market and comparing animals at the same body weight (i.e., taking into account differences in age at slaughter) is of greater interest for meat production in different fields [8–12]. Husbandry conditions are adjusted for every fish species and fishes are selected for growth rate [3, 13, 14]. A selection for improved growth rate results in less mature animals. For that reason, it is of great importance to study animals and the musculature considering their maturity [15]. Therefore, comparing divergently selected lines at a constant body weight is highly a relevant biological model to understanding tissue development in relation to growth rate [11]. DNA, RNA and protein content of muscle tissue can be important indicators for fish growth. Bulow [16] described already the possibility to use the ratio of RNA to DNA as an index of growth to examine feeding condition in fish [16]. Additionally, the protein/DNA ratio is an index of cell size [17] because muscle cell size increases in hypertrophic growing fish.

For direct analysis, we compared skeletal muscle of maraena whitefish with the muscle tissue of another salmonid species, the rainbow trout, a very common species in aquaculture and favourite research model. In rainbow trout, many factors regarding growth [e.g. 18], optimal rearing condition [e.g. 19, 20], animal health and welfare [21, 22] as well as gene expression [e.g. 23] are well studied.

For comparison, both species used in this study were bred in an aquaculture facility and well adapted to the local conditions of the Baltic Sea [6]. In a former study, the physical meat quality of both species was compared [24]. It could be shown, that the physical meat quality pointed out by shear force, water holding capacity and colour is very much alike in these both species, but the meat of rainbow trout is much faster affected by post mortem degradation process and therefore the processing should be faster and the consumer should be aware of it [24].

Skeletal muscle not only contributes to physical strength and performance, but also to efficient macronutrient utilization and storage [25]. Like all other salmonids, rainbow trout and maraena whitefish exhibit an indeterminate growth that lasts a lifetime with continuous accumulation of muscles [26]. Hyperplasia (increase in myofibre number) as well as hypertrophy (increase in myofibre size) contribute to muscle growth, depending on species and life stage [27, 28]. In indeterminate growth both processes remain important during adulthood, however, after reaching 50% maximum body length, the process of hyperplasia becomes minor, so that growth is determined by hypertrophy [29, 30].

There exist several forms of skeletal muscles in fish differing in their visual appearance, ontogenetic origin, cellular anatomy/histology and metabolism. White (anaerobic, highly glycolytic, fast twitch) and red (highly aerobic, slow twitch) muscle fibres can be distinguished, whereas pink muscle fibres (highly aerobic, highly glycolytic, fast twitch) represent an intermediate fibre type [31]. The bulk of skeletal muscle in fish consists of white fibres which due to a relatively poor vascularization favor anaerobic glycolysis, a source for fast ATP production [32, 33], being suitable for bursts and strong swimming activity. The white fibres exhibit a low concentration of myoglobin, mitochondria and lipid droplets [31]. Aerobic red muscle is organized as a triangular band along the flank just beneath the lateral line and is highly vascularized, rich in myoglobin, glycogen and lipids and has numerous mitochondria. Red muscle is used for long-term sustained swimming and moderate speed swimming activity [31, 33]. The activity of muscle specific enzymes like lactate dehydrogenase (LDH), creatine kinase (CK), or isocitrate dehydrogenase (ICDH) can provide important information about the metabolism or energy balance of muscle [34, 35]. In most teleost species, the red fibres account for less than 10% of the total skeletal muscle. Pink myofibres are located between the white and red muscle. The contraction profile of pink fibres combines characteristics of red and white muscle fibres; fast contractions with intermediate resistance to fatigue and intermediate speed [31]. The measurement of muscle fibre diameter in cross-sections is a long-established method, which provides useful data on both hyperplasia and hypertrophy [e.g. 36–38]. Myofibre diameter also allows for the identification of fibre types; red fibres showing the smallest diameter. Myofibres express several contractile proteins like specific Myosin heavy chain (MyHC) isoforms which can be used for fibre type characterization [39], intermediate fibres expressing different isoforms.

Myogenic (stem) cells undergo sequential developmental steps including lineage commitment of progenitor cells, several rounds of proliferation, and initiation and maintenance of the myogenic differentiation program [39]. Although the cell biology of myogenesis in the so far studied fish species seem to be distinct from that of mammals, the genes involved in growth regulation are highly conserved [40]. During early stages of myogenic cell development, the transcription factors Myogenic factor 5 (Myf5), paired box 3 (Pax3) and paired box 7 (Pax7) are detected in myogenic precursor cells [41].

The goal of this present study is a deeper evaluation of the dorsal and ventral myotomal mass and the myogenic properties during fattening stage. To this aim, analyses on nucleic acid content, muscle specific enzyme activity, myofibre type distribution and nuclear density as well as myogenic marker expression were conducted as all factors provide important information for muscle growth and keeping conditions in aquaculture. Altogether, the gained results will give a first insight into muscle metabolism of salmonid species at slaughter size.

## 2 Material and methods

### 2.1 Animals and rearing condition

All fishes were provided from a research station for aquaculture, the Institute of Fisheries of the State Research Centre for Agriculture and Fishery Mecklenburg-Vorpommern in Born (Germany). Within this study, samples from adult animals from maraena whitefish (*C*. *maraena*) and rainbow trout (*O*. *mykiss*, strain BORN, [42]) were used, when it had reached the typical sales size. Under this aspect, the fishes were kept under the commercially and economically most efficient aquaculture conditions (in respect to animal welfare), which are also the current aquaculture standards in Germany (Table 1).

**Table 1:** Summary of the rearing conditions of the two studied fish species maraena whitefish (*Coregonus maraena*) and rainbow trout (*Oncorhynchus mykiss*).

	Maraena whitefish (n = 15)	Rainbow trout (BORN strain; n = 15)
**Aquaculture system**	Recirculation aquaculture system (RAS) [7].	indoor flow through system with natural water from adjacent Darss-Zingst Bodden chain
**Total volume**	50 m³ containing 10 round tanks each 3 m³ in size	100 m³ containing 20 round tanks each 5 m³ in size
**Water temperature**	20.9 ± 1.8 °C	seasonal water temperature changes (0.5 to 26 °C)
**Salinity**	0 PSU	3 to 5 PSU
**Lighting**	24 h	natural light conditions except from September to March were artificial lighting was maintained 8 h light–16 h dark
**Age**	2 years	1 year
**Total length**	32.95 ± 0.27 cm	31.11 ± 0.24 cm
**Circumference**	16.00 ± 0.21 cm	19.31 ± 0.26 cm
**Total weight**	327.07 ± 7.59 g	434.93 ± 10.79 g

Water quality was assured by continuous purification and disinfection (drum filter, moving bed bio-filter and UV light). Water parameters including NH^4+^, NO^2-^ and NO^3-^ concentrations were measured weekly, pH value, temperature and oxygen saturation were constantly recorded. Dissolved oxygen was maintained > 8 mg/l and all physical water parameters were kept in optimal range.

### 2.2 Sampling and data collection

Animals were transported alive in a transport box with an additional oxygen provision from the Institute of Fisheries in Born to the Leibniz Institute for Farm Animal Biology (FBN) in Dummerstorf, Germany. This study followed international, national and institutional guidelines for animal treatment and sacrifice and complied with relevant legislation. Fishes were euthanized according to the animal welfare law Directive 2010/63/EU and TierSchG § 4(3) before slaughtering. Fishes were stunned by a beat on the head and were directly killed by bleed cut in the heart as well as cutting of the spinal cord posterior to the head. Morphometric parameters (total length, circumference, and total weight) were measured and samples of the right epiaxial (dorsal) and hypaxial (ventral) muscles were collected for biochemical analyses. In order to obtain a complete cross-section for the histological and immunohistochemical analyses, the samples were taken from the caudal penduncle.

### 2.3 Biochemical analyses

The dorsal and ventral muscle tissue from 10 animals per species, snap-frozen in liquid nitrogen and stored at -80°C. Subsequently, 100 mg of each sample were pestled and homogenized in 0.01 M potassium phosphate buffer containing 1 mM EDTA in order to analyse the supernatant as previously described in Stange et al. (2020) [43]; dilution of samples was adapted accordingly. DNA and RNA amount were measured fluorometrically by using either Hoechst 33528 (Sigma-Aldrich) or SYBR Green II RNA Gel Stain (MoBiTec) and quantified against a calf thymus DNA standard or a calf liver RNA standard (Sigma-Aldrich), respectively [44]. Total protein amount was determined using Pierce^™^ BCA Protein Assay Kit (Thermo Fisher Scientific, Inc., USA) according to manufactures instructions. The supernatant of samples was as well used to quantify the activity of specific muscle enzymes. The CK-NAC-Hit kit (IFCC method, BIOMED Labordiagnostik GmbH) was used to measure activity of creatine kinase (CK). The activity of lactate dehydrogenase (LDH) and isocitrate dehydrogenase (ICDH) were assessed by measuring NAD(P)H conversion using a NADPH standard curve. All amounts and activities are given per g muscle.

### 2.4 Histological analyses

Since the red region of the muscle is mainly present in the lateral region, for histological analysis there was no need to distinguish between ventral and dorsal muscles. Histological slices of the cross-section of the postanal area were made from six animals of each species. Tissue was shock frozen for 1 min in a combination of precooled isopentane and liquid nitrogen. Afterwards, samples were stored at -80°C. 10 μm thick serial transversal sections were prepared at the cryostat microtome (MC 1950) at -20°C and mounted on slides. Slices were stained via Haematoxylin and Eosin (H&E) as well as NADH-diaphorase staining by routine procedures [45]. For illustration, histological stainings, were imaged with a 100x and 200x magnification using the microscope BZ II 9000 (Keyence Corp, Osaka, Japan). For analysis of the histological samples, sections were photographed via microscope Olympus BX43 using 100x and/or 200x magnification and Olympus camera UC30 and analysed with the software Cell^D (Olympus, Germany) by a pen counter. Using NADH-diaphorase staining, the fibre area was measured. The fibre size distribution was analysed by measuring in average 305±29.7 (S.E.M.) fibres per region. For the examination of the cell nuclei, H&E staining was used to count the cell nuclei. For the red region, all nuclei within a 0.6–0.8 mm^2^ area and in the white region, approx. 1.3–1.7 mm^2^ were counted and extrapolated to the total area size.

### 2.5 Immunohistochemical analyses

For immunhistochemical staining frozen tissue sections were fixed with 4% PFA for 10 min at room temperature (RT) and washed twice with phosphate-buffered saline (PBS) for 5 min. Slices were incubated in blocking buffer (5% bovine serum albumin (BSA), 0.5% Triton X-100 in PBS) for one hour at RT. Sections were incubated with primary antibodies: rabbit polyclonal anti-Pax3 antibody (1:400, abcam ab180754) or rabbit polyclonal anti-Myf5 antibody (1:200, abcam ab125301) in PBS overnight at 4°C. Slices incubated with mouse monoclonal anti-Pax7 antibody (1: 200; abcam ab199010) overnight were treated before staining in 3% H_2_O_2_ in methanol solution to inhibit endogenous peroxidase activity and then blocked with 10% goat serum in PBS for 1 hour. After primary antibody staining, the slices were washed three times in PBS for 5 min and incubated with the secondary antibody (Alexa Fluor 594 goat anti mouse IgG H&L (1:500, abcam ab150116) and Alexa Fluor 488 goat anti rabbit IgG H&L (1: 500, abcam ab150077) or Alexa Fluor 594 goat anti rabbit IgG H&L (1:1000, abcam ab150080) for 90 min at RT. After rinsing with PBS twice for 5 min, sections were mounted with Roti Fluor Care DAPI (Roth, HP20.1). Fluorescence samples were imaged using a Leica DM400B fluorescence microscope at 100x magnification in combination with Leica DFC320 camera and pictures were analysed by software Cell^D by counting the stained and unstained nuclei with a pen counter. For nuclei count, 1.0–1.8 mm^2^ of the red region and in the white region, approx. 2.1–2.8 mm^2^ were analysed.

### 2.6 Statistical analyses

Data are presented as mean ± S.E.M. (Standard error of the mean). For statistical analysis of interfibre and interspecies comparision two-tailed Student´s t-test, two sited ANOVA with Tukey´s test for multiples testing was performed with SAS software version 9.4 (Statistical Analysis institute Inc., USA) for the comparison of ventral and dorsal tissue as well as red and white muscle tissue within a species as well as for the interspecies comparison of the same muscle areas and type. Significant differences was determined as p < 0.05.

## 3 Results

### 3.1 Nucleic acid and protein content and enzyme activity in muscle

**Fig 1:**
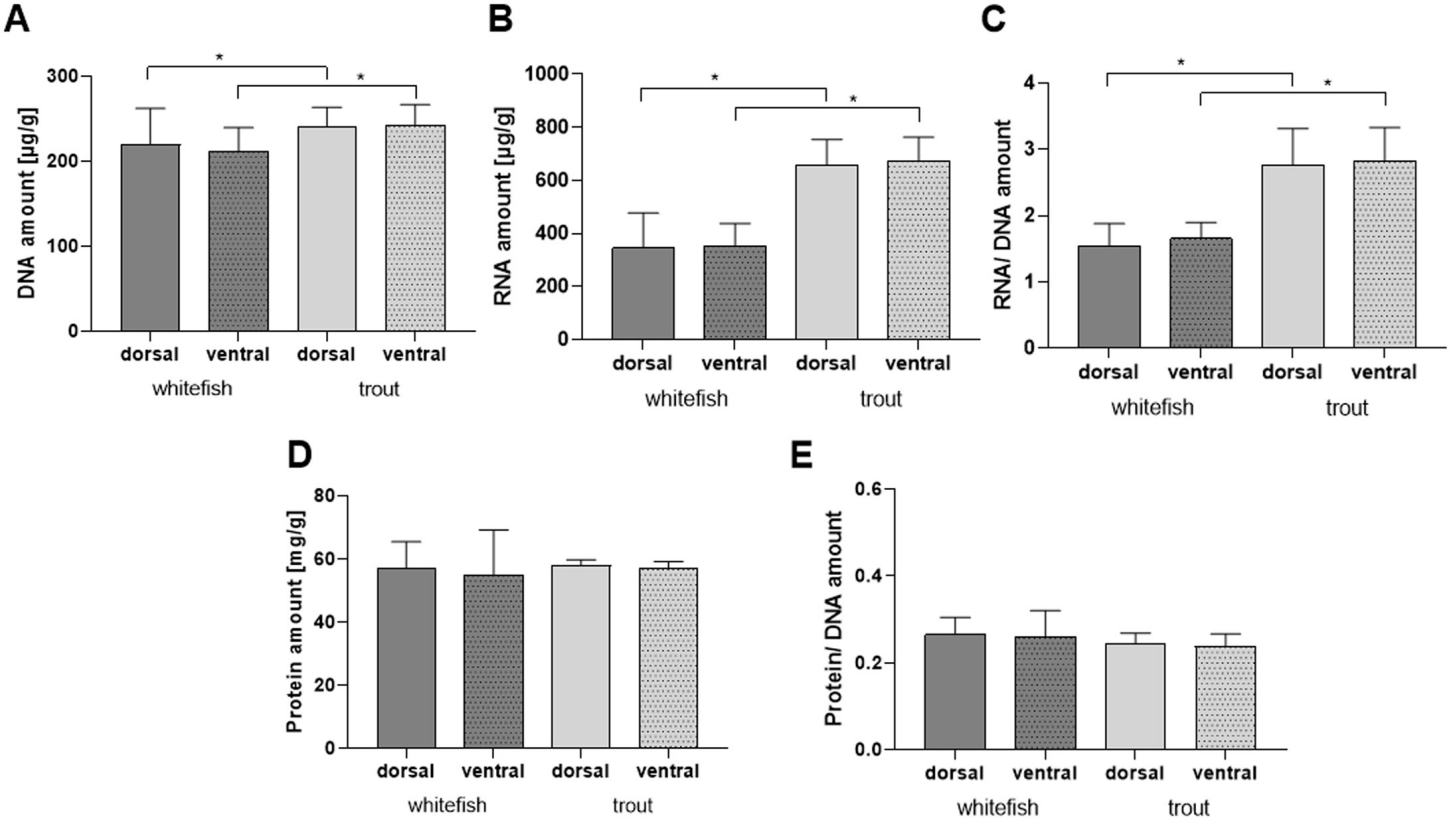
Biochemical analysis of white muscle from dorsal and ventral region. Muscle tissue of maraena whitefish and rainbow trout was analysed to quantify the total amount of DNA (A), RNA (B), and protein (D). Additional the ratio of RNA/ DNA amount (C) and protein/DNA amount (E) is illustrated. For statistical analysis two-tailed Student´s t-test (SAS 9.4) was performed within the species and for interspecies comparison of the same muscle type. * p<0.05 showed significant differences.

**Fig 2:**
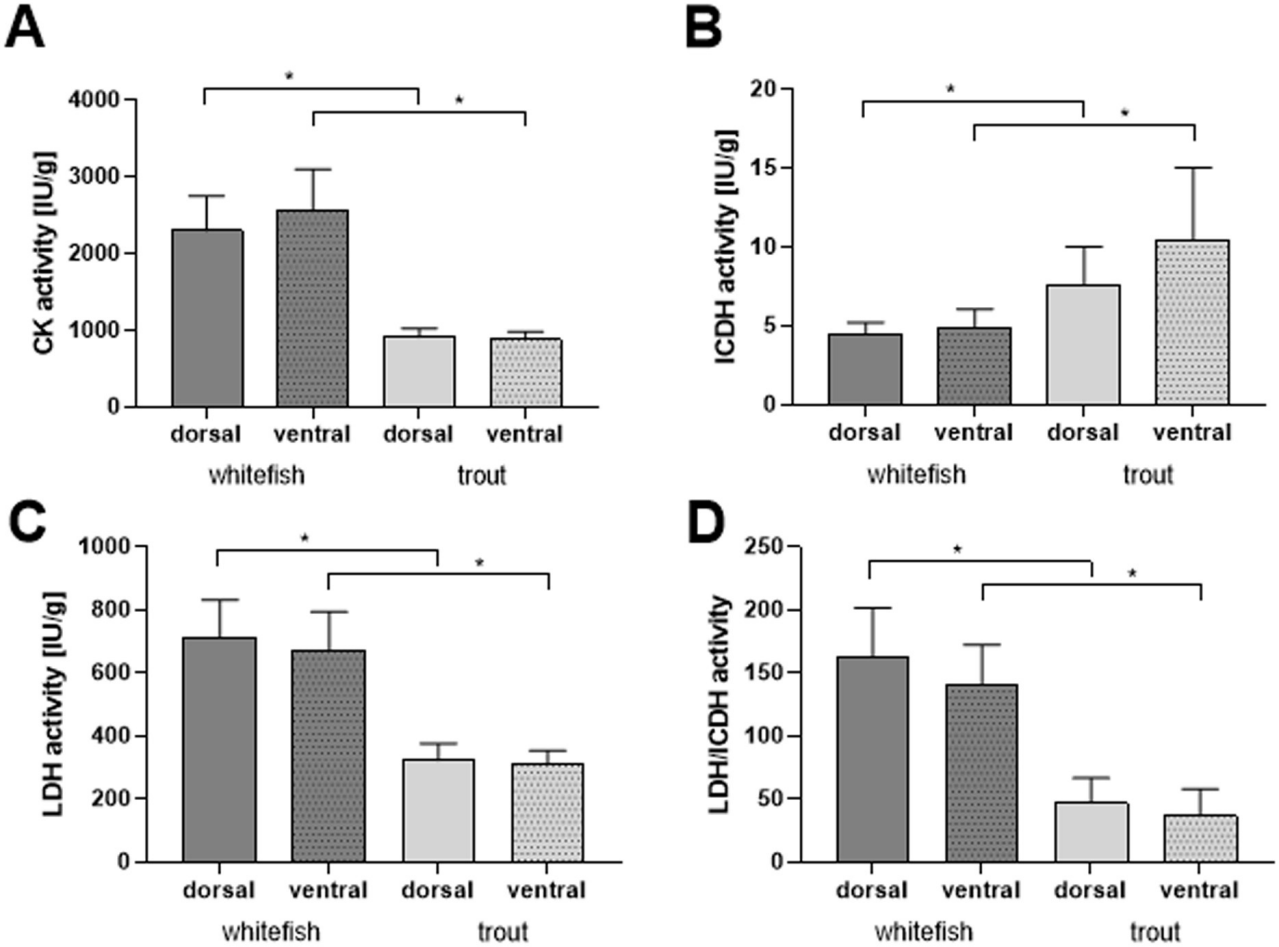
Enzymatic activity analysis of white muscle from dorsal and ventral region. Muscle tissue of maraena whitefish and rainbow trout was analysed to quantify the activity of creatine kinase (CK)—(A), isocitrate dehydrogenase (ICDH)–(B) and lactate dehydrogenase (LDH)–(C). Ratio of LDH/ ICDH activity is illustrated in (D). For statistical analysis two-tailed Student´s t-test (SAS 9.4) was performed within the species and for interspecies comparison of the same muscle type. * p<0.05 showed significant differences.

Muscle tissue samples from maraena whitefish and rainbow trout from the dorsal and ventral region were investigated regarding nucleic acid and protein amount (Fig 1), as well as the activity of muscle-specific enzymes (Fig 2). In all analyses, dorsal and ventral muscle showed the same characteristics in both species. We did find interspecies differences in the DNA and RNA content (Fig 1A & 1B) as well as the enzyme activities (Fig 2). In rainbow trout, the DNA amount was around 9% in dorsal and 13% in ventral muscle higher than in maraena whitefish (Fig 1A) and the RNA amount around double compared to the values found in maraena whitefish muscle tissue (Fig 1B). Due to these values also the RNA/DNA ratio was double in rainbow trout (Fig 1C). Analysis of the protein content revealed similar mean values of around 57 mg/g of total muscle tissue in all four experimental groups (Fig 1D).

Enzymatic activity did show interspecies differences as well (Fig 2). For CK and LDH enzymes, significantly higher activities were measured in dorsal as well as ventral muscle gained from maraena whitefish (Fig 2A & 2C) compared to muscle tissue from trout. In contrast, the ICDH activity was significantly higher in ventral (10.48 ± 1.44°IU/g *vs*. 4.98 ± 0.35°IU/g) and dorsal muscle tissue (7.61 ± 0.76°IU/g *vs*. 4.99 ± 0.21°IU/g) of rainbow trout compared to maraena whitefish (Fig 2B). Due to the significant differences in ICDH and LDH activity, the same significant differences were present when calculating the LDH/ICDH ratio (Fig 2D). It should be further noted that CK activity is 100 to 500-fold higher than ICDH activity.

### 3.2 Analysis of muscle fibre types and nuclear density

**Fig 3:**
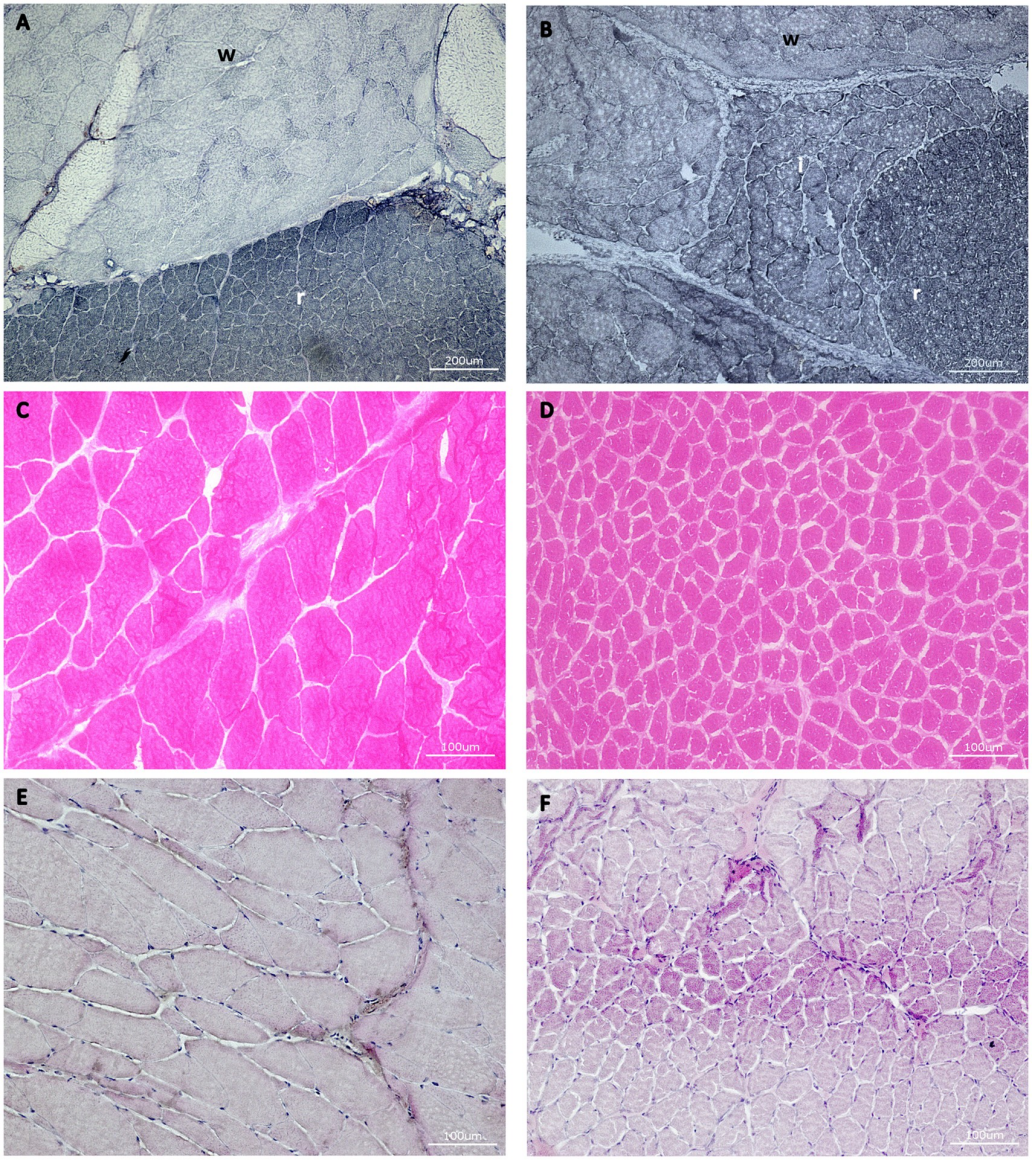
Morphological structure of a cross section of the skeletal muscle. Visualization of white (w), intermediate (i) and red (r) muscle in maraena whitefish (A) and rainbow trout (B) with NADH-Diaphoresis staining. (C-F) Representative illustration of the analysis of the sections from maraena whitefish. Hematoxilin/ staining of (C) white muscle and (D) red muscle as well as Eosin staining of (E) white muscle and (F) red muscle.

In a next step, muscle fibre type composition and area as well as nuclear density were analysed using histological stainings (Fig 3). The most obvious difference was the presence of intermediate fibre area in rainbow trout, which was not present in maraena whitefish (Table 2, Fig 3A and 3B). Rainbow trout had an area of 4,764 ± 152 μm^2^ of white fibre, 3,839 ± 336 μm^2^ of intermediate fibre and 1,532 ± 96 μm^2^ of red fibre. The sizes of the white and red muscle areas were found to be comparable in maraena whitefish. In rainbow trout, the white fibres had the highest area size with nearly 71% of the cells being greater than 50 μm in diameter (in maraena whitefish: 63%) (Table 2). In the intermediate fibre area, 5% fewer cells were in this cell diameter class (Table 2). In contrast to these both muscle types, 75% of the cells of the red fibre area had only a size of 20–50 μm and only 23% of the cells were larger than 50 μm in diameter. In red muscle tissue of maraena whitefish even more cells (82%) exhibited a diameter of 20–50 μm, whereas only 18% were larger than 50 μm. The distribution of fibre size diameter of 20–50 μm and >50 μm depended significantly on the fibre area (white/intermediate/red) or the species (mareaena whitefish vs. rainbow trout), but there was no interaction effect of fibre and species (Table 2).

**Table 2:** Histological analysis of fibres of cross sections.

		fibre area (μm^2^)	average diameter (μm)	smallest diameter (μm)	largest diameter (μm)	% diameter <20 μm	% diameter 20–50 μm	% diameter >50 μm
**Mareana whitefish**	**white fibre area**	4,507 ± 330	68.7 ± 2.9	19.7 ± 0.7	152.3 ± 5.1	0.6 ± 0.3	36.2 ± 4.0	63.3 ± 4.0
	**intermediate fibre area**	0	0	0	0	0	0	0
	**red fibre area**	1,558 ± 53	44.0 ± 0.7	22.0 ± 1.9	61.7 ± 1.5	0.2 ± 0.1	81.6 ± 3.4	18.2 ± 3.4
**Rainbow trout**	**white fibre area**	4,765 ± 153	71.9 ± 1.3	20.3 ± 1.2	150.3 ± 2.9	0.5 ± 0.2	28.8 ± 1.6	70.8 ± 1.7
	**intermediate fibre area**	3,839 ± 336	64.4 ± 3.2	21.9 ± 1.0	148.7 ± 5.5	0.6 ± 0.6	33.6 ± 4.2	65.8 ± 4.6
	**red fibre area**	1,532 ± 96	42.8 ± 1.4	17.4 ± 2.5	71.2 ± 4.6	1.8 ± 1.3	75.5 ± 6.4	22.7 ± 5.7
***p value* Effect of**	**species**	0.107	0.097	0.490	0.000	0.343	0.003	0.003
	**fibre**	0.000	0.000	0.539	0.000	0.729	0.000	0.000
	**species and fibre interaction**	1.000	1.000	0.064	1.000	0.147	1.000	1.000

Results are shown as mean ± S.E.M. Significant differences (p < 0.05) discriminated by species effect, fibre effect and the interaction of species x fibre calculated by ANOVA with Tukey´s test in SAS 9.4.

The analysis of nuclei did not show significant differences between maraena whitefish and rainbow trout. In both species, the number of nuclei per fibre cross section was nearly identical in white and red muscle. There were also no significant differences in the number of nuclei per μm^2^ of muscle type, between whitefish and rainbow trout (Table 2). The analysis revealed considerable differences between the white and the red fibre areas. In the red fibres significantly more nuclei and also a significantly higher density of nuclei were found compared to the white fibres. The number of nuclei found per μm^2^ in white muscle was four times higher than in the red muscle fibres (Table 3).

**Table 3:** Analysis of nuclei in histological cross sections.

			Counted nuclei	nuclei per fibre	Nuclear density (ratio nuclei/ μm^2^ fibre area)
**Maraena whitefish (n = 6)**	White muscle	mean ± S.E.M.	637 ± 32	2.1 ± 0.2	2,127 ± 88
	Red muscle	mean ± S.E.M.	1,054 ± 54	2.5 ± 0.1	619.8 ± 22
	white vs. red	*p*	* < 0.0001	0.115	* < 0.0001
**Rainbow trout (n = 5)**	White muscle	mean ± S.E.M.	728 ± 53	2.0 ± 0.2	2433 ± 191
	Red muscle	mean ± S.E.M.	1,181 ± 95	2.4 ± 0.2	607 ± 16
	white vs. red	*p*	* 0.003	0.182	* < 0.0001
**Whitefish *vs*. Trout**	White muscle	*p*	0.16	0.72	0.16
	Red muscle	*p*	0.26	0.72	0.664

P-values analysed by two tailed Student´s t-test (SAS 9.4).

* p<0.05 showed significant differences.

### 3.3 Analysis of myogenic precursor cells

**Fig 4:**
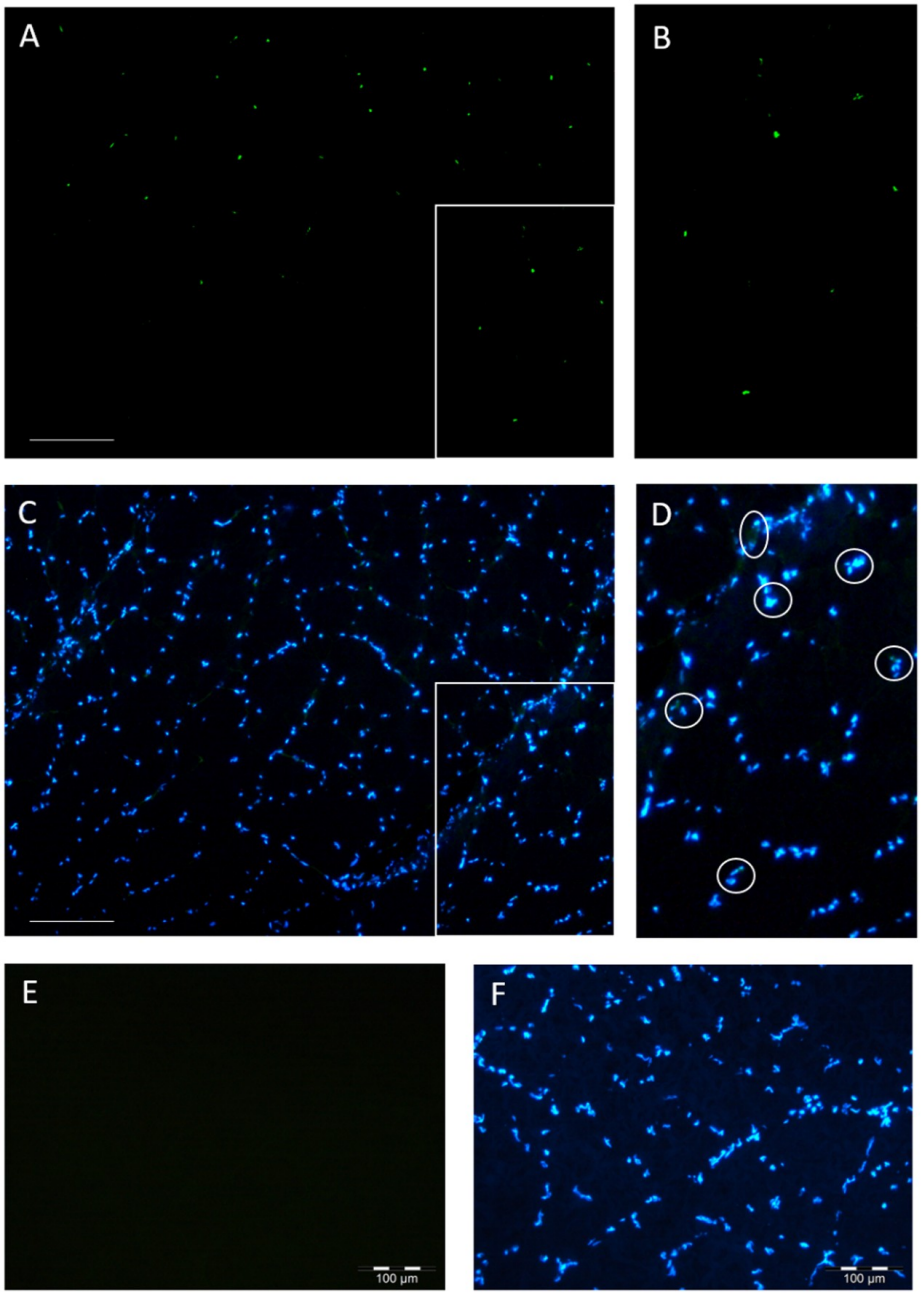
Immunhistochemical staining of a cross section of the skeletal muscle. Exemplary illustration of the antibody stainings against Myf5 for stem cell localization in sections from maraena whitefish of white muscle (A—in B is shown a section). Cell nuclei staining with DAPI (C—in D is shown a section). E & F—negative control. Scale 200 μm.

The number and distribution of Pax3^+^, Pax7^+^, and Myf5^+^ cells were analysed as a proportion of total nuclei in a next step (Fig 4, Table 4). The intraspecies comparison of the red and white muscle revealed that significant differences were present. In both species, the red muscle had a higher content of stem and progenitor cells. For example, the percentage amount of Pax7^+^ cells in red muscle of maraena whitefish was 0.82% ± 0.23 (vs. white muscle: 0.03% ± 0.02) and in rainbow trout 1.13% ± 0.13 (vs. white muscle: 0.26% ± 0.12). The comparison between the species showed that their muscles are much alike regarding the expression of the selected markers. However, a significantly higher proportion of Myf5^+^ cells was found in red muscle of rainbow trout (11.63% ± 1.02) vs. maraena whitefish (7.18% ± 1.17)), (Table 4). Additionally, there was a trend that rainbow trout exhibit more Pax7^+^ cells and maraena whitefish more Pax3^+^ cells within the white muscle (Table 4).

**Table 4:** Presence of Pax3, Pax7 and Myf5 positive cells analysed by immunohistostaining in marena whitefish (*Coregonus maraena*) and rainbow trout strain BORN (*Oncorhynchus mykiss*).

	Maraena whitefish			Rainbow trout			Whitefish *vs*. Trout	
	White muscle	Red muscle	white *vs*. red	White muscle	Red muscle	white *vs*. red	White muscle	Red muscle
	mean ± S.E.M.	mean ± S.E.M	*p*	mean ± S.E.M	mean ± S.E.M	*p*	*p*	*p*
Number total nuclei	1,265 ± 121	2,623 ± 470	* 0.04	1,467 ± 142	2,531 ± 429	* 0.05	0.31	0.89
number Pax3^+^	10.4 ± 3.1	23.8 ± 5.8	0.07	4.2 ± 1.0	32.6 ± 9.2	* 0.037	0.09	0.44
% Pax3^+^	0.8 ± 0.2	0.9 ± 0.2	0.72	0.3 ± 0.1	1.3 ± 0.3	* 0.041	0.085	0.37
Number total nuclei	1,233 ± 52	2,566 ± 115	* <0.001	1,522 ± 62	2,447 ± 277	* 0.03	* 0.01	0.704
number Pax7^+^	0.4 ± 0.2	21.0 ± 6.2	* 0.03	4.2 ±2.0	26.8 ± 2.8	* <0.001	0.09	0.42
% Pax7^+^	0.03 ± 0.02	0.8 ± 0.2	* 0.03	0.3 ± 0.1	1.1 ± 0.1	* 0.001	0.09	0.267
Number total nuclei	1,321 ± 84	2,473 ± 65	<0.0001	1,585 ± 160	2,567 ± 223	* 0.012	0.16	0.66
number Myf5^+^	109 ± 9	177 ± 27	* 0.06	175 ± 13	297 ± 35	* 0.02	* 0.004	* 0.029
% Myf5^+^	8.3 ± 0.9	7.2 ± 1.2	0.46	11.5 ± 1.6	11.6 ± 1.0	0.94	0.12	* 0.03

Results are shown as mean ± SEM. P-values analysed by two tailed Student´s t-test (SAS 9.4).

* p<0.05 showed significant differences.

## 4 Discussion

Within this study, we examined ventral and dorsal muscle tissue of two different salmonid species at commercial slaughter size to compare similar and practically relevant body weights, although different growth rates were seen. This comparison is common in fish but also in other farmed animal species. When assessing welfare and production traits in slow and fast growing animal groups (genotypes), scientists aim to compare similar body weights and therefore these animals are grown until this goal is achieved [46–50]. It is challenging to compare muscle characteristics of growth-selected lines or species with different growth rates because animals will possibly not reach the same degree of maturity when reaching a predefined body weight or age. Nevertheless, comparing animals with the same body weight is a priority since carcass weight is fixed by the market. Improved growth rate enables faster turnover in production, and this creates economic benefits in terms of reduced fixed and variable costs per kg fish produced [49] and it is also highly biologically relevant due to the interrelation between growth rate and tissue development [51].

To reach this aim, muscle characteristics like RNA, DNA, and protein content, as well as muscle fibre size and type within the muscle tissue of rainbow trout and maraena whitefish were analysed as well as the cells´ presence, size and myogenic marker expression. Furthermore, we examined muscle specific enzyme activity in order to take a first look at the metabolic cycle through which muscle growth takes place.

### 4.1 Hypertrophic growth process in whitefish and trout

Rainbow trout and maraena whitefish, the two salmonid species studied here, reach slaughter size very quickly. However, considerable growth differences are present within the salmonids. To compare muscle tissue characteristics of both species, the typical sale size of around 30 cm body length was chosen. Since the size of the animals is crucial for the food industry, the goal is to achieve these sizes in as little time as possible whereas the influences of this selection process on muscle tissue has not been investigated in detail in fish. The trout reached sale size after already one year whereas the whitefish needed two years for reaching the same size. Within this study, both fish species showed nearly identical total length, although in trout the circumference was significantly larger and with 25% more weight also significantly heavier than maraena whitefish. These morphological parameters indicate a faster growth of rainbow trout under the investigated conditions. Results can be explained by different domestication status of both species. Rainbow trout breeding programs for several decades lead to well adapted aquaculture strains with fastest growth rates combined with phenotypic characteristics and vitality. Contrary, adaptations of maraena whitefish for intensive aquaculture generated only from a few generations focus still on optimal rearing conditions, healthy fish stock and optimization of artificial breeding. Fast growing breeding lines will be selected in the next years on this base.

In a next step, we investigated whether the rapid growth seen in both salmonid species was mainly caused by hyperplasia or hypertrophy. The presence of small, newly formed muscle fibres is an indicator for hyperplastic growth [31]. In contrast, during hypertrophic growth, the size of existing muscle fibres notably increases. In fishes, length of muscle fibres decreases in length towards the tail end of the fillet [31]; and the muscle fibre cross sectional area is smaller at the tail and head than in between [31, 46]. For this reason, cell size and its percentage amount are used as an appropriate indicator for fibre size. As in other fish, in both species, the red muscle is superficially located just under the skin, while the white muscle is located further in. Overall, the muscle mass makes up around 60% of the fish’s mass, with at least 70% of the muscle in all fish being attributed to the white muscle fibres [32]. The present analyses revealed that the absolute area of red and white muscle area was comparable between trout and maraena whitefish. Fibre diameters did not differ significantly between trout and whitefish within white or red fibre area. Notably, the mean diameter and also the largest diameter found was considerably higher in white muscle than in red muscle pointing to a more pronounced hypertrophic growth within this area. In maraena whitefish, 74% of the muscle area was composed of white muscle and 26% of red muscle area; no intermediate fibres were found. Contrastingly, in the trout intermediate fibres were seen in addition to the white and red muscle layers. This intermediate (pink) layer was 20% smaller than the white area. Pink muscle behaves similarly to white muscle and is therefore also defined as an “intermediate white muscle” [47]. It can be speculated that the presence of intermediate muscle fibres contributes to faster growth in trout, possibly by dynamic counteracting of hyperplasia and hypertrophy. White and intermediate fibre area, both showing high cell diameters, accounted for 85% of the muscle area in trout, whereas only 15% of the muscle area was formed by red muscle. Taking into account the muscle fibre distribution, the trout had a slightly higher amount of large cells than the whitefish. These findings on the one hand correspond to the larger circumference and larger muscle mass of the trout. On the other hand, having larger muscle areas with high diameter fibres, is related to a more pronounced hypertrophic growth in trout.

Additionally, a significantly higher DNA and RNA content was seen in trout than in maraena whitefish muscle tissue. Consequently, the RNA/DNA ratio in trout ventral and dorsal muscles was double compared to the whitefish. Several studies in recent years have shown that the cytoplasm/DNA ratio can vary during hypertrophic growth and therefore the DNA content is not a fixed factor of fibre size [52, 53]. It was also described that the DNA content increases with cell size [54, 55], both parameters being elevated in the trout muscle investigated here. This is supported by the results of the cell nucleus analysis. A slightly higher number of total nuclei in both red and white tissue in trout was documented.

Taken together, the larger body circumference and the proportional reduction in red muscle area accompanied by the increase in DNA content and number of cells with high diameter argue for a higher degree of hypertrophic growth in trout compared to whitefish.

### 4.2 Energy metabolism in muscle tissue

Naturally, trout are adapted to running water and the red muscle propels the fish at relatively high speeds. The pink or “intermediate white muscle”is not active during low swimming speeds in aquaculture [47], but becomes active at a much higher speed, perhaps when blood oxygen starts to become limiting, and this requires a higher degree of anaerobic metabolism from this species [47]. The oxygen limitation going along with anaerobic metabolism dominating in white muscle might also be related to the subtle reduction of nuclei per muscle fibre compared to red muscle found in our study. It is well known, that the red muscles used for continuous swimming during slow and moderate speeds generate their energy from aerobic mitochondrial processes, whereas the fast white muscles used for the so-called burst swimming get their energy mainly from glycolysis [56]. Therefore, white muscle used for swimming at maximum speed through the fastest possible acceleration, for example to catch prey or escape [32], also tire more quickly [57]. In the present study, the activity of muscle specific enzymes was determined on the white muscle due to the small amount of red muscle. LDH and ICDH are well known parameters for anaerobic, glycolytic or aerobic, oxidative (alactic) metabolism, respectively [58]. For ATP production, LDH plays an important role within the glycolytic cycle and is therefore crucially important to maintain muscle physiology. ICDH is additionally associated with high muscle activity (contributes to muscle relaxation and contraction) and muscle growth [59]. A drastically higher LDH activity (up to 140 fold vs. ICDH) in trout and maraena whitefish reflects an anaerobic, glycolytic metabolism laid out for fast energy release like it is needed in white muscle. LDH activity depends on many physiological factors and seems to be very different among fish species. For example, in cyprinids lactate begins to accumulate in muscle tissue at relatively modest swimming speeds [60, 61], whereas in the rainbow trout lactate production commences in a burst-like fashion only at high swimming speeds [62]. Wieser et al. [63] measured within *Coregonus sp*. very high LDH activities in the white muscle which the authors explained by their sprinting properties [63]. Our data support the statement that the trout was more actively developing at the time of measurement, since ICDH values were significantly higher in trout muscle. Nevertheless, anaerobic metabolism was clearly favoured in both investigated species.

### 4.3 Molecular characteristics of cells in fish muscle tissue

For hypertrophic growth, myofibres need to merge and myonuclei contribute to these fibres [64, 65]. These nuclei mostly are obtained from satellite cells, which are present in the muscle within their niche between sarcolemma and basal lamina in adults [29]. Due to the lifelong muscle growth of indeterminate growing fishes, there is a persistent need for nuclei.

Pax3/7 and Myf5 expression is found in satellite cells committed to the myogenic lineage as well as in myoblasts [66, 67] and Pax3/7 are also present in PICs (PW1+ interstitial cells) [68]. During the recent years, several studies showed that Pax3/7 play a key role in fibre maintenance and are upregulated during hyperplasia in fishes with lifelong growth, like the salmonids [67, 69, 70]. In order to gain a first insight into the molecular *status quo* of the cells within fish muscle tissue, we selected these three crucial muscle specific markers for analysis. Pax3, Pax7, and Myf5 could be successfully detected in histological sections, however the percentage of positive cells was rather low. The vast majority of cells did not express one of the selected markers. Both species were mostly comparable regarding myogenic marker expression. Both markers are downregulated during myogenic differentiation process and higher levels are indicative for a higher amount of non-differentiated cells.

In accord with that, significantly lower CK values were found in trout muscle compared to maraena whitefish. Whereas the enzyme is not present in myoblasts, CK will be activated within terminal differentiation of myocytes and can be used as a measure for myogenic differentiation [71]. Elevated CK values in muscle tissue of maraena whitefish argue for an accelerated differentiation process. In conjunction with a lower expression of the early myogenic markers Pax7 and Myf5 this might reduce capacities for (hypertrophic) muscle growth.

This study investigates the muscle characteristics of two fish species when reaching market sale size (slaughter size) reared under commercially used conditions. At carcass weight, the skeletal muscle of these fishes have not been studied in detail until now and previous studies used different methods, complicating the comparison to our findings. The holistic approach shown here allows for a first comparison under practical considerations by including the potential influences of age and growth conditions (e.g. water temperature) on muscle development. The faster growth rate of the trout under the investigated and commercially used rearing conditions makes this species popular and successful as an aquaculture fish. In the future, detailed studies will have to clarify the specific influence each of the aquaculture parameters on growth (kinetics) and identify optimal possibilities for modulation. To unravel underlying mechanisms for differing growth rates and muscle properties is a prerequisite to optimize aquaculture conditions in a species-specific manner. Further investigations on cell properties, including analyses of additional myogenic markers, and their energy metabolism and differentiation potential are needed in the future, as well.
